# Modern Food Systems Challenged by Food Safety Culture

**DOI:** 10.17113/ftb.64.01.26.9508

**Published:** 2026-02-15

**Authors:** Mojca Jevšnik Podlesnik, Peter Raspor

**Affiliations:** 1 Department of Sanitary Engineering Research, Faculty of Health Sciences, University of Ljubljana, Slovenia; 2 Emeritus Professor of Microbiology and Biotechnology, University of Ljubljana, Slovenia

**Keywords:** food safety, human factor, food safety culture, human behaviour, food systems, good practices

## Abstract

Despite decades of regulatory development, standardized food safety management systems, and technological advances, foodborne outbreaks, recalls, and food fraud continue to pose significant public health and societal challenges. These persistent failures increasingly reveal systemic vulnerabilities that deficiencies in legislation or formal control mechanisms alone cannot explain. Instead, they highlight the critical role of human behaviour, organizational culture, and socio-technical interactions within modern, complex agrifood networks. Food safety culture has therefore emerged as a key determinant of food safety performance, linking regulatory frameworks with everyday practices in food establishments. While HACCP-based systems clearly define procedures and responsibilities, their effectiveness is limited when behavioural consistency, leadership commitment, communication, and resource availability are weak. Research consistently shows that even well-designed systems are insufficiently monitored when organizational alignment and behavioural adherence are lacking, allowing deviations from safe practices to persist. Contemporary approaches move beyond compliance-driven models towards cultural transformation, emphasizing leadership engagement, effective risk communication, learning-oriented environments, and evidence-based behavioural interventions. Increasingly, digital tools and real-time monitoring systems support this transition by strengthening feedback, transparency, and adaptive risk management across food systems. Strengthening food safety culture therefore requires coordinated, multi-level action that integrates governance, technology and human-oriented approaches. Such transformation is essential not only for improving food safety outcomes but also for protecting public health, maintaining consumer trust and enhancing the long-term resilience and sustainability of modern food systems.

## INTRODUCTION

Food safety is one of the most critical public health challenges of our time. Foodborne diseases constitute a substantial global public health burden. It is estimated that each year approx. 600 million people worldwide fall ill after consuming contaminated food, resulting in around 420 000 deaths annually. In addition to health impacts, foodborne diseases impose considerable economic and social costs, particularly in low- and middle-income countries (*1*). Although technological advances and regulatory frameworks have significantly improved food safety systems, the human element remains the most vulnerable component in the food safety chain. In Europe, the latest data from the European Food Safety Authority (EFSA) (*2*) reveal concerning trends. In 2023, 148 181 campylobacteriosis cases were reported, an increase from 139 225 in 2022. After campylobacteriosis, salmonellosis was the second most reported gastrointestinal infection in humans, with 77 486 cases compared to 65 478 cases in 2022 (*2*). In 2023, listeriosis cases reached their highest level since 2007, while campylobacteriosis and salmonellosis remained the most frequently reported zoonotic diseases in the EU (European Centre for Disease Prevention and Control (ECDC)) (*3*). This trend is particularly concerning given Europe's ageing population, as elderly individuals face higher risks of severe symptoms from foodborne illnesses. When chemical and physical hazards are added to the microbiological dimension of food safety, we face a truly multifaceted challenge for the future global food supply.

### Systemic vulnerabilities

These persistent challenges reveal a fundamental paradox: despite increasingly sophisticated regulatory frameworks and technological capabilities, food safety incidents continue to occur at alarming rates. This paradox reflects the complex interplay between biological hazards, such as microbial adaptation and survival mechanisms, and the socio-technical dynamics that have characterized contemporary food systems for some time (*4*, *5*) and have become even more intense in since World Food Safety Day was established (*6*). Food safety failures rarely result from a single cause; rather, they arise from the intricate interaction of human behaviour, organizational culture, technological systems, and regulatory pressures. Understanding these dynamics is essential for identifying systemic vulnerabilities – the weak points where formal compliance diverges from actual practice, where communication breaks down across supply chain nodes, and where economic pressures compromise safety protocols. These vulnerabilities are not merely technical issues to be resolved, but are symptomatic of deeper cultural and organizational patterns that institutional frameworks alone cannot address. The evolution of food safety governance reflects this growing recognition of complexity. From the early days of command-and-control regulation focused on end-product testing, we have moved towards preventive, system-based approaches embodied in frameworks like Hazard Analysis and Critical Control Point (HACCP) system (*7*), and more recently, risk-based verification systems that integrate transparency and sustainability principles (*8*, *9*).

However, the implementation of these legislative frameworks within contemporary agrifood networks reveals significant gaps. Modern food supply chains are characterized by unprecedented complexity: globalized sourcing, multiple intermediaries, rapid product turnover, and diverse stakeholder ecosystems. In this context, traditional regulatory approaches that assume linear, hierarchical control structures often prove inadequate. The challenge is no longer simply to ensure compliance with prescribed standards, but to foster adaptive capacity and collective responsibility across decentralized, networked organizations (*10*).

This reality requires a fundamental shift from the institutionalization of food safety culture to genuine cultural transformation within food systems. Institutionalization – the establishment of formal policies, procedures, and compliance mechanisms – is necessary but not sufficient for sustainable food safety. True cultural transformation involves moving beyond procedural compliance with the internalization of food safety values at all organizational levels, development of proactive rather than reactive mindsets, and fostering learning cultures that continuously adapt to emerging risks. This transformation must bridge the gap between the ’work-as-imagined’ in regulations and procedures and the ’work-as-done’ in actual food handling environments, addressing the socio-technical realities that create systemic vulnerabilities while operationalizing food safety principles in ways that resonate with the distributed, networked nature of contemporary agrifood systems (*11*).

## FROM INSTITUTIONALIZATION OF FOOD SAFETY CULTURE TO CULTURAL TRANSFORMATION IN FOOD SYSTEMS

Despite sophisticated HACCP systems and advanced food processing technologies, human behaviour consistently emerges as the primary risk factor in food contamination incidents (*12*). This vulnerability appears at multiple stages of the food production and consumption chains, often linked to attitudes specific to different professions along these chains (*13*). For more than two decades, the food industry has operated under the paradigm of comprehensive supply chain oversight, captured in slogans such as ’From farm to fork’ and ’From stable to table’ (*14*). However, structural and political changes have not adequately supported the essence of this development. The persistent gap between regulatory ambition and operational reality reveals a fundamental truth: institutional frameworks alone cannot guarantee food safety (*15*). Despite assurances from proponents of structured systems that technical controls would suffice, operational challenges were evident from the early implementation phases in different stages of food supply chain (*16*-*18*).

The most crucial element remains, and will continue to be, the human factor, with all its rational and emotional characteristics that dictate behaviour in practical circumstances at each step of food production, processing, distribution, preparation, and delivery. This recognition marks a critical shift from viewing food safety culture as merely an institutional requirement to understanding it as a dynamic, live reality that must be actively cultivated and sustained (*19*).

### The emergence of food safety culture as a critical research domain

The emergence of food safety culture reflects a broader shift from purely technical and compliance-based approaches towards recognising human and organizational factors as central determinants of food safety performance (*20*, *21*). This conceptual transition has recently been formalized within the European regulatory framework through Commission Regulation (EU) 2021/382 (*8*), which amended Regulation (EC) No 852/2004 by explicitly introducing food safety culture as a mandatory component of food business operations.

By requiring food business operators to establish, maintain and provide evidence of an appropriate food safety culture, the regulation represents a significant regulatory milestone. It moves beyond traditional hazard control and documentation towards expectations related to leadership commitment, employee awareness, communication, and shared responsibility for food safety outcomes (*22*-*24*). Importantly, the regulation acknowledges that formal food safety management systems alone are insufficient if they are not supported by consistent behaviours and organizational alignment in everyday practice (*15*, *25*).

However, while Regulation (EU) 2021/382 (*8*) provides regulatory legitimacy to the concept of food safety culture, it deliberately leaves its operationalization open to interpretation. The absence of prescriptive criteria, standardized indicators, or validated assessment tools places responsibility for implementation largely on food business operators and competent authorities. This regulatory flexibility allows context-specific adaptation but also introduces challenges related to consistency, monitoring and enforcement across diverse food systems, including short food chains (*11*, *26*, *27*).

Consequently, the regulation reinforces the need for interdisciplinary approaches that integrate regulatory compliance with behavioural science, organizational learning, and leadership practices. Food safety culture thus emerges not merely as a regulatory requirement, but as a dynamic socio-technical construct linking governance frameworks with human behaviour and organizational performance (*28*).

### The behavioural dimension of food safety is complex and multifaceted

The consistency, accuracy and correctness with which employees perform defined tasks and work procedures are influenced by multiple interacting factors: individual knowledge and motivation, competence levels, understanding of food safety principles, hygienic awareness, attitude towards work, job satisfaction, self-efficacy, and the availability of temporal, human, and material resources (*12*, *29*-*31*). From a broader organizational perspective, food safety culture also depends on the effectiveness of HACCP-based management system implementation, company policy, management commitment and leadership quality, employee awareness, communication patterns, work environment characteristics, resource availability, and the processes of risk factor identification and assessment. In this context, risk management must remain dynamically responsive to a rapidly evolving global environment marked by the continual emergence of new challenges and conflicts (*22*, *25*). This requirement reflects not transient concern but an enduring, structural challenge.

### The persistent challenge: from knowledge to practice

Past research clearly demonstrates that, despite continuous education efforts, food safety assurance systems remain insufficiently monitored and imperfectly controlled due to inherent risks associated with the human nature of work (*30*-*33*). This reveals a critical distinction between knowing what should be done and consistently doing it. Food safety assurance fundamentally depends on how individuals who handle food behave in ways that represent minimal risk to food products and, consequently, to human health (*20*, *22*).

Training is an important social element in bridging this knowledge-practice gap, ensuring that necessary information is correctly understood by users (*34*) and effectively applied in practice (*33*, *35*). Food business operators must ensure regular, ongoing training of employees (*11*, *36*). However, training alone is not sufficient. The learning process must influence individual behaviour to create reliable and aware workers who conscientiously perform their food safety tasks (*12*, *22*, *30*, *36*, *37*). This transformation from trained employee to intrinsically motivated food safety practitioner is the essence of cultural change (*20*, *22*).

## FROM COMPLIANCE TO CULTURE: THE EVOLUTION OF FOOD SAFETY STANDARDS

Food business operators today navigate a complex landscape of requirements that extends far beyond mandatory legislative compliance. In addition to legal obligations, many organizations implement additional requirements defined in various national, international and private standards. These voluntary commitments aim to increase employee awareness and knowledge, improve control of risk factors in food handling, and ensure quality and safe products (*38*).

However, the voluntary nature of these enhanced standards reveals an important cultural divide. For food business operators with a strong food safety culture, implementing these standards is an ambitious, self-motivated pursuit of excellence. For those with a weaker culture, implementation is often imposed externally. This external imposition has emerged primarily due to changes in business practices and power dynamics within food supply chains, reflected in contractual requirements where buyers make business relationships with producers conditional on establishing certified standards (*29*, *39*, *40*).

### The rise of private label products has further complicated this landscape

Some retailers now order food products under their own brands from specific producers, effectively transferring the responsibility for producing safe and high-quality food from the producer or supplier to the retailer or buyer (*41*). To protect their reputation and brand equity, retailers often require stricter and more comprehensive preventive measures for private label products than legislation mandates, thereby raising the bar for food safety assurance across their supply chains. This market-driven elevation of standards has prompted in-depth research to identify weak points that could endanger food production from quality or safety perspectives (*36*, *42*).

### The institutionalization paradox

This evolution of standards and requirements creates a paradox: the very institutionalization of food safety culture through proliferating standards, certifications, and audits may inadvertently undermine the development of genuine cultural transformation (*43*). When food safety culture becomes primarily a matter of documented compliance, i.e. checkbox exercises, audit preparation, and certification maintenance, it risks becoming disconnected from the daily experiences of food handlers and the intrinsic values that should guide their behaviour (*44*).

Genuine cultural transformation requires moving beyond this institutionalized approach. Organizations must shift from extrinsic motivation, such as avoiding penalties and passing audits, to intrinsic motivation characterized by personal commitment to food safety. This transformation also demands moving from procedural compliance to adaptive expertise that enables employees to respond effectively to novel situations. Furthermore, companies need to transition from top-down enforcement approaches to distributed ownership of food safety outcomes across all organizational levels. Finally, organizations must evolve from static documentation systems to dynamic learning systems that continuously improve and adapt to changing circumstances. The challenge facing contemporary food systems is therefore not simply implementing more rigorous standards or conducting more frequent audits, but rather fostering authentic cultural change that embeds food safety values deeply within organizational DNA and individual professional identity. This transformation must bridge the persistent gap between work-as-imagined in regulations and standards and work-as-done in the complex realities of daily food handling operations (*22*, *23*). This paradox is one that the European Union is actively attempting to address through intensive educational initiatives aimed at strengthening the competencies of professionals working within governmental food safety structures across Member States (*45*, *46*).

### The role of human behaviour in food safety

Food legislation provides a legal framework for implementing regulations that guide and manage risks and ensure food safety throughout the entire food chain (*47*). This significantly affects public health (*48*) and defines the responsibilities of food business operators, supervisory bodies and consumers (*40*, *49*).

Today, foodborne infections and poisoning still account for a significant proportion of illnesses, with viral foodborne diseases becoming increasingly prominent (*2*, *16*). Contributing factors include the development of novel food products designed to meet consumer demand for innovation, often based on traditional substrates but produced, handled, or consumed in new ways that introduce additional safety challenges (*50*).

Foodborne viral infections remain a significant public health concern due to their interaction with changes in food systems and consumption practices. Evidence shows that viruses are increasingly associated with minimally processed and ready-to-eat foods, traditional raw materials used in novel product formats, and consumption contexts that reduce the effectiveness of conventional control measures. These risks are amplified by globalized food supply chains, increased consumption of food prepared outside the home, reduced use of preservatives, demographic shifts towards more vulnerable populations, and persistent gaps in food handling knowledge at the household level, allowing foodborne viruses to persist despite existing controls (*51*). The implementation of the HACCP system for food safety assurance has significantly improved the control of risk factors, including chemical and physical hazards (*52*). HACCP is a food safety assurance system based on preventive measures to identify and control hazards (*11*, *53*). However, other aspects must also be considered, such as food safety culture, which is reflected in employee behaviour when working with food (*24*, *40*, *54*). The HACCP system can be even more effective with certification procedures of voluntary Global Food Safety Initiative (GFSI) group standards (*44*), but only if these systems are embedded within company policy and translated into well-organized daily practices in food establishments (*55*) as well as in food handling practices at home (*56*).

Employees in food business establishments play a crucial role in ensuring food safety (*57*). Some studies have identified demographic characteristics as reasons for errors and mistakes when working with food (*34*, *48*, *58*), lack of time, money and other resources (*59*, *60*), workplace pressure, competence, motivation and employee satisfaction (*35*, *37*, *61*). Observation of employees working with food has shown that although they demonstrate sufficient knowledge about food safety assurance and related standards, they do not always apply this knowledge in practice (*33*, *60*, *62*, *63*). Therefore, it is very important for food companies to establish a high level of food safety culture that promotes correct implementation of work procedures (*22*). This depends on elements of the food safety climate among employees such as leadership, communication, commitment, risk awareness and resource availability as well as employee competence and education level, including food donation system (*64*-*66*).

Studies have shown that the knowledge, attitudes, and practices of food handlers are important factors in preventing foodborne illness (*67*). However, research consistently reveals significant gaps between theoretical knowledge and practical application. Food handlers may understand basic food safety principles but fail to implement them consistently due to time pressure, inadequate resources, complacency or language barriers (*68*).

Appropriate leadership encourages and guides employees to implement hygienic measures and food safety procedures in accordance with business goals, vision and company standards. Proper communication about food safety ensures the transfer of practices, knowledge and all relevant information from management, through middle management, to those who handle operational tasks with food. Commitment to food safety helps raise the values of all employees and strengthens their conviction in the correctness of food safety procedures, which must align with company policy and goals (*15*, *20*). This must consider the realistic perception of the seriousness of risk factors (*57*). Employees may be aware of risks but do not control them for various reasons, which Griffith et al. (*20*) call ’optimistic bias’ and ’illusion of control’.

De Boeck et al. (*64*) state that food safety culture consists of two conceptual aspects, namely human and technical-managerial, which ultimately result in safe and high-quality food. The human aspect includes organizational and individual levels. It involves an interaction of food safety climate elements, manifested in employee behaviour when working with food, while the technical-managerial aspect reflects the implemented food safety system in the company with existing control and activities (*65*).

Fatimah et al. (*57*) argue that strengthening food safety culture requires consistent implementation of food safety policies across all hierarchical levels, fostering collaboration between departments and generations of employees, establishing a reliable system for evaluating work performance, and ensuring effective communication about relevant risk factors. Such risk factors have been identified not only in declarations (*69*), but also within legislation and regulatory frameworks, as well as in the practical, day-to-day realities of food handling environments (*70*).

### Psychological and behavioural factors in food safety

Human decision-making in food safety contexts is significantly influenced by various cognitive biases that can compromise safety outcomes (*20*), which is well observed during food safety days (*71*). Optimism bias leads individuals to believe that foodborne illness happens to others rather than themselves, creating a false sense of security that may result in neglecting proper safety protocols (*20*, *57*). This psychological tendency is compounded by familiarity bias, where people assume that familiar foods are inherently safe regardless of how they are handled or processed. Additionally, the availability heuristic causes individuals to overestimate the risks of highly publicized food safety incidents while underestimating more common but less newsworthy risks, leading to misallocated attention and resources in safety management (*33*).

The organizational culture within food establishments plays a crucial role in shaping individual behaviour and safety outcomes (*20*, *64*). Environments that consistently prioritize productivity over safety often create conditions where risky behaviours become normalized, establishing systemic vulnerabilities throughout the operation (*37*). When organizations fail to demonstrate genuine commitment to food safety through their policies, resource allocation and daily practices, employees are more likely to adopt shortcuts and compromise safety standards, particularly under time pressure or when facing competing priorities (*54*, *57*, *59*).

Traditional food safety training programmes often fail to achieve their intended behavioural outcomes due to several fundamental limitations (*31*, *33*, *72*). Most conventional training approaches focus primarily on knowledge transfer rather than genuine behaviour change, assuming that increased awareness will automatically lead to improved practices (*34*, *60*, *72*). These programmes often lack practical application opportunities that would allow participants to practise new skills in realistic settings, and typically fail to address workplace-specific challenges that employees encounter in their daily operations (*61*). Furthermore, many training programmes do not adequately consider cultural and linguistic diversity among workers, potentially excluding important segments of the workforce from effective safety education (*60*, *72*, *73*).

Contemporary research has identified several evidence-based solutions that address these psychological and organizational challenges more effectively (*65*). Behavioural intervention strategies have shown promise, with nudging techniques involving environmental modifications that naturally promote safer behaviours without relying solely on conscious decision-making (*74*). Social norm interventions leverage peer influence to encourage compliance by making safe behaviours more visible and socially desirable within the workplace (*57*). Real-time feedback systems provide immediate monitoring and correction of unsafe practices, allowing for prompt behavioural adjustments before problems escalate (*62*, *65*).

Enhanced training approaches represent another critical avenue for improvement (*72*). Competency-based training shifts the focus from theoretical knowledge to demonstrable skills, ensuring that participants can perform safety procedures correctly rather than simply understanding them conceptually (*60*, *72*). Scenario-based learning uses realistic situations to help workers practise decision-making skills in controlled environments, building confidence and competence for real-world applications (*63*). Continuous reinforcement through regular refresher training and ongoing competency assessments helps maintain high safety standards over time rather than allowing skills to deteriorate after initial training (*60*, *72*).

Technology integration offers further opportunities to strengthen food safety culture through digital monitoring systems that provide automated temperature and time tracking, reducing reliance on manual processes prone to human error (*75*). Mobile training platforms make safety education more accessible and personalized, allowing workers to learn at their own pace and in their preferred language (*74*). Predictive analytics can identify high-risk situations before contamination occurs, enabling proactive interventions rather than reactive responses to safety failures (*76*).

Finally, developing organizational culture requires sustained commitment to leadership visibility in supporting safety priorities, creating systems that empower employees to report safety concerns without fear of retaliation, and implementing recognition programmes that reward safe behaviours and safety improvements (*20*, *63*). These comprehensive approaches recognize that effective food safety culture requires addressing both individual psychological factors and broader organizational dynamics that influence behaviour in complex, interconnected ways (*22*).

Nudge tools offer a subtle yet effective method for improving hygiene behaviour among employees in the food industry. Štefančič and Jevšnik (*77*) conducted a case study in a retirement home, testing the effectiveness of different nudges such as storytelling about foodborne outbreaks, a thermometer image, citrus scent, and citrus scent combined with a sign on hygiene criteria. The findings show that storytelling alone had little effect, while the thermometer image significantly improved compliance with critical control points. The citrus scent combined with a sign markedly improved behaviour at all key stages of food preparation, whereas citrus scent alone had mixed effects, ranging from relaxation to distraction.

Behavioural economics research further demonstrates that nudge tools based on priming (e.g. signs, words, sensory cues) and affective triggers can influence behavioural change among food handlers. A consistent finding is that knowledge-based training alone is often insufficient to ensure sustained compliance, whereas nudge-based interventions can significantly enhance hygiene behaviour by targeting automatic responses and habitual practices. This highlights that interventions shaping choice architecture and reducing cognitive load may outperform approaches that rely primarily on deliberate decision-making (*78*, *79*).

Similarly, a systematized review (*80*) found that priming nudges (sensory or verbal cues), affective salience nudges (emotional triggers like disgust or appeal), messenger nudges (social norm framing), and default nudges (pre-set safer or healthier options) consistently improved food choice behaviour. Importantly, priming was effective in most cases, demonstrating the power of subtle environmental signals in shaping everyday hygiene and food-related practices. These insights underline the psychological principle that reducing cognitive load and making safe or desired behaviours the easiest choice increases compliance and sustainability of behavioural change. In addition to classical psychological and behavioural determinants such as knowledge, attitudes, leadership and organisational climate, contemporary organisational practice increasingly recognises the role of operational philosophies and emerging technologies in shaping employee behaviour and motivation. Lean manufacturing, with its emphasis on continuous improvement and respect for people, has been shown to influence organisational culture, employee engagement, and motivational dynamics by embedding efficiency-oriented and participatory behaviours in everyday work practices (*81*).

More recently, the integration of digital technologies, particularly Artificial Intelligence (AI), into lean-oriented organisations has introduced new psychological and behavioural dynamics that may affect employee performance and motivation. Empirical evidence suggests that AI adoption within lean systems can enhance employee engagement when AI-enabled tools are perceived as supportive of human decision-making, autonomy, and skill utilisation (*82*). In these contexts, AI may reduce repetitive workload, improve role clarity, and strengthen perceived competence, all of which are well-established drivers of motivation and performance.

However, the behavioural and psychological effects of AI are not uniformly positive. When AI is perceived as a mechanism of surveillance, control, or a threat to job security, it may increase stress, resistance, and demotivation among employees. Recent studies on AI-enabled job characteristics highlight that the impact of AI on employee well-being and performance depends strongly on implementation strategies, transparency, and the extent to which human-centred principles are maintained (*83*). These findings suggest that both lean manufacturing and AI should be considered important contextual factors that interact with traditional psychological and behavioural determinants, particularly in organisational settings where employee behaviour plays a critical role in safety-related performance.

## FROM LEGISLATION TO CONTEMPORARY AGRIFOOD NETWORK OPERATIONALIZATION

### The paradigm shift: from compliance to culture

The transition from compliance-based food safety systems to a genuine food safety culture marks a fundamental paradigm shift in how organizations approach risk prevention. This shift is not merely conceptual but responds to the changing nature of food systems themselves, from linear, hierarchical production chains to complex, networked ecosystems of suppliers, processors, distributors and retailers (*26*). Traditional compliance relies on external enforcement, audits and documentation, mechanisms rooted in command-and-control regulatory models that assume direct oversight and hierarchical authority structures. However, these mechanisms often fail to ensure consistent behaviour when direct supervision is absent, particularly in the distributed, multi-nodal environments characteristic of contemporary agrifood networks (*79*, *84*).

Recent studies highlight that food safety culture emphasizes shared values, internalized responsibility, and proactive engagement across all organizational levels (*20*, *22*). In networked food systems, this cultural dimension becomes even more critical, as food safety outcomes depend not only on individual organizational performance but also on collective coordination across multiple autonomous actors (*43*). Leadership plays a critical role in this process, as managers must move beyond ’box-ticking’ compliance to foster ownership, communication, and continuous learning (*85*). Research also shows that a strong food safety culture correlates with improved hygiene behaviour and reduced non-compliance, since employees are more likely to ’do the right thing when no one is watching’ (*28*). Thus, the evolution from compliance to culture represents not only regulatory alignment but also a sustainable strategy for risk management and organizational resilience in increasingly complex supply chain environments.

### Barriers and enablers in cultural transformation

Translating legislative intent into operational reality across contemporary agrifood networks faces significant structural challenges. Pai et al. (*85*) emphasize that major barriers to establishing a positive food safety culture include limited resources, difficulties in risk communication, and challenges in behavioural change. These barriers are strengthened in networked settings, where information must cross organizational boundaries, resource constraints vary dramatically between large retailers and small suppliers, and cultural norms and practices differ across geographic and organizational contexts (*31*, *84*).

Nickell and Hinsz (*86*) and Manning (*87*) highlight the critical role of leadership and organizational commitment in fostering effective food safety cultures, noting that food safety culture has transitioned from a narrow compliance-based concept to a comprehensive organizational value essential for ensuring food safety. However, in contemporary agrifood networks, leadership must function at multiple levels: within individual organizations, across supply chain partnerships, and at the network level, where collective governance mechanisms shape behaviour (*88*). This multi-level leadership challenge requires new forms of coordination and shared accountability that go beyond traditional buyer-supplier relationships (*89*).

### Contemporary trends: investment, technology and behavioural science

Recent evidence suggests that behavioural interventions combining nudging strategies with knowledge-based approaches can effectively improve food safety practices, highlighting the importance of moving beyond purely informational frameworks (*74*). These metrics show that food safety culture is not merely an ethical or regulatory imperative but a strategic business advantage in competitive food markets.

Contemporary approaches to food safety culture increasingly emphasize behavioural interventions and digital tools that enable large-scale operationalization across distributed networks. A proven method to improve frontline employee engagement in effective food safety behaviours is the concept of ’nudging’ (*48*, *90*, *91*), behavioural interventions that guide choices without restricting options (*78*). In networked food systems, digital platforms enable nudging interventions to be deployed consistently across multiple sites and organizations, creating standardized behavioural frameworks even without direct supervision (*79*). Regulatory frameworks increasingly recognize the potential of digital tools to support food safety management systems, facilitating monitoring, documentation and compliance processes (*75*).

The digitalization of food safety management represents a crucial link between legislative frameworks and network-level operations. Digital traceability systems, real-time monitoring technologies, blockchain-based verification, and data analytics platforms offer new possibilities for transparency, accountability, and rapid response across complex supply chains. These technologies provide what traditional legislation and compliance mechanisms could not: visibility into ’work-as-done’ rather than merely ’work-as-documented’, early warning systems that detect emerging risks before they become incidents, and feedback loops that support continuous learning among network participants (*92*, *93*).

### System level determinants of food safety culture

Research developments in food safety culture show a shift from traditional compliance-based approaches to more comprehensive strategies for behavioural and organisational change. Recent developments increasingly emphasize the role of digital technologies, such as data-driven decision support, digital incentives and tailored feedback systems, in shaping food-related behaviors. These approaches support continuous improvement processes by enabling context-sensitive interventions and adaptive learning mechanisms in modern food safety management systems (*91*). However, implementing these strategies in contemporary agrifood networks requires additional dimensions beyond individual organizational culture (*94*). As contemporary agrifood systems become increasingly interconnected, the effective implementation of food safety culture initiatives can no longer rely solely on transformations within individual organizations. Instead, it requires a broader systems perspective that recognizes the interaction between organizational culture, governance structures, information flows, and adaptive capacities across the wider supply network. In this context, several additional dimensions are critical for translating food safety culture principles into practice across complex, interdependent food-system structures. Network-level governance mechanisms must complement organizational culture by establishing shared norms, mutual expectations, and collective accountability among supply chain participants. These mechanisms include collaborative standard setting, joint auditing and shared investment in food safety infrastructure that benefits all network members (*95*).

Information architecture must enable transparent communication and knowledge sharing across organizational boundaries to address critical vulnerabilities in the food supply chain. Food safety incidents often result from information asymmetries or communication failures between supply chain nodes, and digital platforms combined with standardized data protocols can effectively mitigate these vulnerabilities (*96*).

Adaptive capacity must be distributed throughout the network rather than concentrated in the hands of a few powerful actors. Small- and medium-sized enterprises, which form the backbone of many food supply chains, require targeted support and resources to implement food safety culture initiatives that correspond to their capabilities and operational contexts.

Regulatory frameworks must also evolve to recognize and actively support network-level approaches to food safety. Traditional food safety legislation focuses primarily on individual business operators, but contemporary regulation should facilitate collaborative governance arrangements, incentivize information sharing across organizational boundaries, and create enabling conditions for collective learning throughout the supply chain (*97*).

The challenge of operationalizing food safety culture in contemporary agrifood networks therefore extends beyond implementing standards or deploying technologies within individual organizations. It requires fundamentally rethinking how food safety is governed, monitored and improved within complex, dynamic systems where risks emerge from interactions among multiple actors, technologies and environments. This network-oriented approach represents the next frontier in the evolution from legislation to real-life practice in food safety assurance (*43*).

Table 1 summarizes key short-term and long-term actions needed to strengthen food safety culture across seven critical organizational domains. Short-term measures focus on establishing basic behavioural expectations, communication clarity, and initial cultural diagnostics, while long-term actions emphasize systemic integration, leadership development, and data-driven organizational learning. Together, these measures illustrate how food safety culture evolves from operational compliance towards a mature, strategically embedded organizational capability.

**Table 1:** Short-term vs long-term actions for strengthening food safety culture

Element (7 key areas)	Short-term actions (1–4 years)	Long-term actions (5–10 years)
1. Leadership and commitment	Managers model safe behaviour daily; ensure resources and clear expectations.	Build leadership development programs; implement food safety culture into strategic governance and accountability systems.
2. Communication and awareness	Standardize internal communication; clarify rules, reminders, and visual cues; align messages with suppliers.	Develop global communication frameworks across multicultural supply chains; embed two-way communication practices.
3. Training and competency	Provide frequent, task-specific micro-trainings focused on behaviour, not just knowledge.	Create long-term competency frameworks supported by behavioural science; integrate digital learning ecosystems.
4. Behaviour and workplace practices	Address immediate gaps between knowledge and actual behaviour; introduce simple behaviour checklists.	Implement continuous behaviour monitoring, coaching, and incentives; build a culture where safe behaviour is habitual.
5. Reporting and transparency	Introduce non-punitive reporting of near misses and unsafe acts to increase openness.	Develop a mature learning organization where reporting data is analyzed and used to predict and prevent failures.
6. Assessment and monitoring	Use basic culture surveys, interviews and observations to identify weak points.	Integrate the culture of key performance indicators (KPIs) into audits and certifications; adopt digital, real-time monitoring tools and analytics.
7. Supply-chain alignment and systems integration	Define clear expectations for suppliers, contractors, cloud kitchens, gig workers.	Build harmonized international standards and fully integrated food safety culture requirements throughout global supply chains.

## COMPILATION OF CURRENT FOOD SAFETY MANAGEMENT PERSPECTIVE

The role of food safety culture in shaping risks within modern food systems is significant. While humans represent the weakest link in food safety systems, they also hold the greatest potential for improvement. This paradox encapsulates the central challenge facing contemporary food safety governance: how to transform individual human vulnerability into collective systemic resilience. Accordingly, food safety must be understood not only as a technical and regulatory domain but also as a fundamentally social and cultural phenomenon shaped by collective behaviours, organizational dynamics, and the broader context in which food systems operate (*42*, *98*).

Food safety is a fundamental human right, yet billions of people worldwide remain at risk from unsafe food. The persistent gap between regulatory ambition and food safety outcomes, evidenced by increasing foodborne illness rates even in highly regulated environments, demonstrates that traditional approaches cantered on compliance and technical controls are necessary but insufficient.

Addressing human behaviour in food safety requires moving beyond the reductionist view of humans as error-prone components to be controlled towards recognizing people as adaptive agents whose behaviour emerges from complex interactions between individual characteristics, organizational cultures, technological systems and socio-economic pressures (*26*, *99*). This requires a multifaceted approach that combines scientific understanding of behavioural psychology with practical interventions tailored to the specific contexts of food production, processing, and distribution. Critically, it demands acknowledging the socio-technical nature of food safety failures: incidents rarely result from isolated human errors but from systemic vulnerabilities where organizational pressures, resource constraints, communication breakdowns, and cultural norms create conditions in which errors become likely or inevitable. Empirical evidence supports this system-oriented perspective, demonstrating that lower maturity of food safety culture is associated with higher costs of quality, reflecting inefficiencies, rework and failure-related losses that stem from underlying organizational and cultural weaknesses rather than individual misconduct (*100*).

Food practices arise from culture, history and human behaviour; technical controls alone do not guarantee safety. Modern systems must therefore treat the human and social aspects as central, not peripheral (*43*, *91*).

### From institutionalization to transformation

Success in reducing human-related food safety risks depends on moving beyond the institutionalization of food safety culture, i.e. the establishment of formal policies, standards, and compliance mechanisms, towards genuine cultural transformation. As this analysis has shown, the proliferation of food safety standards, certifications, and audit requirements may paradoxically undermine authentic cultural change when they become ends in themselves, rather than embedding food safety values deeply within organizational practice and professional identity (*11*).

Genuine transformation requires creating supportive organizational cultures that foster intrinsic motivation rather than relying solely on external enforcement, implementing evidence-based behavioural interventions that recognize the context-specific nature of food handling work, developing adaptive expertise to respond to novel situations rather than merely ensuring procedural compliance, and establishing learning systems that bridge the persistent gap between work-as-imagined in regulations and work-as-done in actual operational environments (*25*).

This transformation must recognize that sustainable food safety improvements require addressing the complex interplay between individual knowledge and competence, organizational systems and leadership, technological capabilities and constraints, and the broader social, economic, and regulatory influences that shape behaviour across supply chains (*62*).

There is a persistent gap between knowledge and safe behaviour among food handlers. Our surveys and studies repeatedly show that knowledge, attitudes and self-reported practices do not always align; training alone often fails to change daily behaviour without cultural support (*30*, *31*, *33*, *36*).

### Operationalizing culture in networked food systems

The challenge of cultural transformation is further complicated by the networked nature of contemporary agrifood systems. Food safety outcomes increasingly depend not only on individual organizational performance but also on collective coordination among multiple autonomous actors: farmers, processors, distributors, retailers, and food service operators, each operating under different pressures, resources and cultural contexts. Traditional regulatory frameworks designed for hierarchical, linear production chains are inadequate for governing these complex, dynamic networks. Operationalizing food safety culture in this context requires multi-level interventions: network level governance mechanisms that establish shared norms and collective accountability across supply chain participants; information architectures that enable transparent communication and knowledge sharing across organizational boundaries; distributed adaptive capacity that supports small and medium enterprises in implementing food safety improvements appropriate to their contexts; and evolved regulatory frameworks that facilitate collaborative governance and create enabling conditions for collective learning rather than focusing solely on individual compliance (*97*, *101*).

The digital transformation of food safety management through traceability systems, real-time monitoring, data analytics, and behavioural nudging platforms offers unprecedented opportunities to operationalize food safety culture at scale across distributed networks. However, technology alone cannot create culture; digital tools must be designed and deployed in ways that support rather than undermine human agency, expertise, and intrinsic motivation (*90*).

Food safety culture is multidimensional and measurable, but complex. Research on dimensionality demonstrates that culture comprises several factors (leadership, communication, risk awareness, resources and routines) and that measurement requires rigorous, context-specific tools (*101*).

### Modern food system trends increase the cultural challenge

Contemporary food systems are undergoing rapid transformation driven by globalization, digitalization, and changing consumption patterns. These developments have fundamentally altered how food is produced, processed, distributed and consumed, increasing both structural complexity and behavioural demands across the food chain. Globalized sourcing and extended supply chains reduce direct oversight, increase heterogeneity in practices and standards, and amplify coordination challenges between geographically and culturally diverse actors. As a result, food safety increasingly depends on shared values, consistent behaviours, and effective communication across organizational and national boundaries rather than on centralized control alone (*75*, *101*).

At the same time, digital transformation has introduced new operational models such as e-commerce platforms, home delivery services, cloud kitchens, and so-called ’virtual restaurants’, where food is prepared, handled and distributed outside traditional, physically co-located establishments. These models often involve fragmented responsibilities, high staff turnover, algorithm-driven work organization, and limited face-to-face supervision, all of which place additional strain on the development and maintenance of food safety culture. In such contexts, formal procedures and documentation may exist, but their consistent enactment relies heavily on employees’ internalized commitment to food safety principles (*85*, *100*, *101*).

Further challenges arise from the introduction of novel ingredients, alternative proteins, minimally processed foods, and innovative processing technologies, which may outpace existing regulatory frameworks and organizational learning processes. When technological and market innovation advances faster than cultural adaptation, gaps emerge between work-as-imagined in food safety systems and work-as-done in daily operations. These gaps heighten the risk of safety practices becoming inconsistent, especially under economic pressure, time constraints, and competitive delivery models (*26*, *101*).

Together, these trends significantly increase the need for robust and adaptive food safety cultures that can function effectively under conditions of uncertainty, decentralization and rapid change. Rather than relying solely on compliance mechanisms, contemporary food systems require cultures that support shared responsibility, learning and resilience among firms and throughout the supply chain, enabling safe practices to be sustained even when traditional supervisory and regulatory controls are limited (*26*, *101*, *102*).

### The path forward: integrated approaches for systemic resilience

The path forward requires collaboration among food safety professionals, behavioural scientists, technology developers, and policymakers to create systems that support and enhance human performance, rather than simply expecting perfection. This integrated approach must address several critical priorities that span research, practice, policy and technology.

Research and practice should focus on understanding and addressing systemic vulnerabilities by examining the organizational, technological, and economic conditions that make errors likely, rather than merely attributing failures to individual human inadequacy (*26*, *101*). This shift in perspective recognizes that human error is often a symptom of deeper systemic issues that require structural solutions.

Investment in workforce development must go beyond basic food safety training to foster professional identity, adaptive expertise, and genuine ownership of food safety outcomes among employees. Evidence demonstrates that such investments yield substantial returns in productivity, employee retention, and overall business performance, showing that food safety culture is not merely an ethical imperative but also a strategic competitive advantage (*85*).

Regulatory innovation must complement existing legislative frameworks with mechanisms that enable learning, collaboration, and continuous improvement in food system networks. This includes creating safe spaces for reporting and learning from near misses without fear of punishment, incentivizing transparency and information sharing between supply chain partners, and recognizing that adaptive capacity is as important as procedural compliance in ensuring food safety (*43*).

Technological development must prioritize human-centred design principles that enhance rather than replace human judgement in critical decision-making processes. Technology should support the development of adaptive expertise rather than deskilling workers through excessive automation, and it must create effective feedback loops that facilitate continuous learning and improvement (*65*).

Addressing food safety culture requires confronting uncomfortable truths about power, resource allocation, and responsibility distribution within food systems. Research on food safety culture determinants consistently demonstrates that behaviour is shaped not only by individual knowledge and attitudes but also by broader organizational, economic and contextual conditions. Small producers and frontline workers, who often operate with limited resources and decision-making power, may therefore carry disproportionate responsibility for food safety outcomes while simultaneously facing economic pressures that constrain their ability to prioritize safety over productivity. Genuine cultural transformation thus requires moving beyond individual-level behaviour change to address the structural conditions and systemic inequalities that fundamentally shape food safety practices across the system (*20*, *23*, *101*).

The challenge of transforming food safety culture is ultimately a challenge of transforming food systems themselves: from compliance-focused, hierarchical structures to learning-oriented, networked ecosystems characterized by shared values, collective accountability, and distributed resilience. Only through this systemic transformation can we move beyond treating humans as the weakest link and recognize and cultivate their potential as the adaptive, intelligent foundation of food safety assurance (Table 2). In doing so, we transform not only how we prevent foodborne illness but also how we understand the agrifood chain through the relationships between people, organizations, technology, and the complex systems that feed the world.

**Table 2:** Key performance indicators (KPIs) that would significantly support incremental improvements in food safety culture

KPI	What it measures	Why it matters
1. Near miss reporting rate	Number of near misses reported per month/employee.	Indicates openness, trust, and a non-punitive reporting culture. More frequent reporting usually means stronger culture.
2. Training completion and competency score	Percentage of staff completing required food safety training and passing competency checks.	Measures not only attendance but actual understanding and application of safe practices.
3. Leadership walkthrough frequency	Number of documented food safety leadership observations/engagements per week or month.	Shows visible leadership commitment and reinforces safe behaviour.
4. Behaviour compliance score	Percentage compliance observed during hygiene, PPE, handwashing, and CCP-related behaviour checks.	Directly reflects whether everyday actions match food safety expectations.
5. Corrective action closure time	Average time from identifying an issue to resolving it.	Demonstrates responsiveness, accountability and operational discipline.
6. Internal communication effectiveness	Percentage of employees who report understanding food-safety messages (*via* short surveys or pulse checks).	Measures clarity, consistency, and reach of safety communication.
7. Supplier or contractor food safety culture compliance	Percentage of suppliers meeting or exceeding defined culture-related requirements (audits, behaviour standards, reporting).	Ensures that food safety culture extends throughout the entire supply chain, not just within the company.

PPE=personal protective equipment, CCP=critical control point

Table 2 shows a set of key performance indicators (KPIs) that enable systematic monitoring and incremental improvement of food safety culture. These indicators capture critical dimensions such as reporting transparency, behavioural compliance, leadership engagement, and the effectiveness of communication and training. Together, they provide organizations with quantifiable metrics that support evidence-based decision-making and strengthen food-safety culture both internally and across the supply chain.

## CONCLUSIONS

Modern food systems are increasingly complex, globalized and technologically dynamic, making food-safety culture a central pillar in ensuring safe food from farm to fork. This review, synthesizing research aligned with international authorities (EFSA, FAO, FDA, GFSI and WHO), demonstrates that contemporary food safety depends not only on technical controls but also on human behaviour, organizational values, and shared responsibility.

A consistent understanding emerges from global literature: even the most advanced HACCP-based systems can fail when individuals do not internalize safe practices, communicate risks effectively, or operate within supportive leadership structures. A persistent gap between knowledge and behaviour remains, highlighting the critical need for continuous, context-adapted education and sustained leadership engagement.

The multidimensional nature of food safety culture, encompassing attitudes, communication, risk perception, resource allocation, and social norms, requires systematic measurement, management and improvement. Contemporary challenges, including globalization, supply chain complexity, outsourcing and e-commerce, amplify the necessity for cultural alignment among all food system actors.

Building and sustaining a robust food safety culture is not merely best practice but a strategic imperative for 21st-century food systems. As global megatrends intensify and food systems continue to evolve, sustained safety can only be achieved through the deliberate integration, continuous monitoring, and adaptive improvement of both technical controls and cultural dimensions at each stage of production and supply networks. Only through this holistic approach can the global food supply remain safe, resilient, and trustworthy for future generations.

## References

[1] Food safety. Geneva, Switzerland: World Health Organization (WHO); 2024. Available from: https://www.who.int/news-room/fact-sheets/detail/food-safety.

[2] European Food Safety Authority (EFSA). The European Union One Health 2023 zoonoses report. EFSA J. 2024;22(12):e9106. 10.2903/j.efsa.2024.9106 39659847 PMC11629028

[3] Zoonotic diseases on the rise in EU: Listeriosis cases hit highest level since 2007. Stockholm, Sweden: European Centre for Disease Prevention and Control (ECDC); 2024. Available from: https://www.ecdc.europa.eu/en/news-events/zoonotic-diseases-rise-eu-listeriosis-cases-hit-highest-level-2007.

[4] Lang T Barling D. Food security and food sustainability: Reformulating the debate. Geogr J. 2012;178(4):313–26. 10.1111/j.1475-4959.2012.00480.x

[5] European food systems in a changing world: Forward look. Strasbourg, France: European Science Foundation, Brussels, Belgium: European Cooperation in Science and Technology (COST); 2009.

[6] Raspor P. Codex Alimentarius and its faces. In: Raspor P, editor. Food standards save lives. 5th World Food Safety Day 2023. Ljubljana, Slovenia: European Declaration on Food, Technology and Nutrition Network; 2023. pp. 16-8

[7] Raspor P, Ambrožič M, Jevšnik M. Food chain safety management systems: The impact of good practices. In: Yanniotis S, Taoukis P, Stoforos N, Karathanos V, editors. Advances in food process engineering research and applications. Food Engineering Series. Boston, MA, USA: Springer; 2013. pp. 607-25. 10.1007/978-1-4614-7906-2_30

[8] Commission Regulation (EU) 2021/382 of 3 March 2021 amending the Annexes to Regulation (EC) No 852/2004 of the European Parliament and of the Council on the hygiene of foodstuffs as regards food allergen management, redistribution of food and food safety culture. OJ L. 2021;74:3-6. Available from: https://eur-lex.europa.eu/eli/reg/2021/382/oj.

[9] Malik S Krishnaswamy K Mustapha A. Hazard analysis and risk-based preventive controls (HARPC): Current food safety and quality standards for complementary foods. Foods. 2021;10(9):2199. 10.3390/foods10092199 34574310 PMC8468952

[10] Su IH Wu L Tan KH. The future of the food supply chain: A systematic literature review and research directions towards sustainability, resilience, and technology adoption. J Digit Econ. 2023;2:303–16. 10.1016/j.jdec.2024.03.001

[11] Raspor P, Jevšnik M, Ambrožič M. Food safety systems. In: Selamat J, Iqbal SZ, editors. Food safety: Basic concepts, recent issues, and future challenges. Cham, Switzerland: Springer; 2016. pp. 3-31. 10.1007/978-3-319-39253-0_1

[12] Griffith CJ Livesey KM Clayton DA. Food safety culture: The evolution of an emerging risk factor? Br Food J. 2010;112(4):426–38. 10.1108/00070701011034439

[13] Raspor P. Food and nutrition as permanent challenge. In: Raspor P, Mrša V, editors. Food, nutrition and environment: Positions in Central European space. Zagreb, Croatia: Croatian Academy of Engineering; 2022. pp. 9-18.

[14] Raspor P. Total food chain safety: How good practices can contribute? Trends Food Sci Technol. 2008;19(8):405–12. 10.1016/j.tifs.2007.08.009

[15] Jevšnik M Bobnar S Sraka Šadl M Raspor P. Food safety culture among food handlers in Slovenia. Acta Microbiol Bulg. 2021;37(1):10–21.

[16] Ambrožič M Božič T Jevšnik M Cook N Raspor P. Compliance of proposed Codex Alimentarius guidelines for virus management with principles of good practice. Acta Aliment. 2011;40(3):364–75. 10.1556/AAlim.40.2011.3.7

[17] Ambrožič M Kukec A Jevšnik M Smole Možina S Raspor P. Food safety expertise among professional food handlers and consumers related to foodborne viruses: Case Slovenia. Int J Sanitary Eng Res. 2016;10(1):4–19.

[18] Jevšnik M Bauer M Zore A Raspor P. Hygienic status of small and medium sized food enterprises during adoption of HACCP system. Int J Food Sci Technol Nutr. 2007;1(1):95–113.

[19] Zabukošek M Jevšnik M Maletič M. Analysis of dimensionality of food safety culture: An empirical examination of a Slovenian food processing company. Int J Sanitary Eng Res. 2016;10(1):20–34.

[20] Griffith CJ Livesey KM Clayton DA. The assessment of food safety culture. Br Food J. 2010;112(4):439–56. 10.1108/00070701011034448

[21] Aybar Espinoza MS Flink C Boisen N Scheutz F Käsbohrer A. Microbiological sampling and analyses in the food business operators’ HACCP-based self-control programmes. Front Food Sci Technol. 2023;3:1110359. 10.3389/frfst.2023.1110359

[22] Yiannas F. Food safety culture: Creating a behavior-based food safety management system. New York, NY, USA: Springer; 2009. 10.1007/978-0-387-72867-4

[23] De Boeck E Jacxsens L Bollaerts M Vlerick P. Food safety climate in food processing organizations: Development and validation of a self-assessment tool. Trends Food Sci Technol. 2015;46(2):242–51. 10.1016/j.tifs.2015.09.006

[24] Behrens JH Vedovato GM Cervato-Mancuso AM Bastos DHM. Social representations of safety in food services. Food Res Int. 2015;74:324–8. 10.1016/j.foodres.2015.05.024 28411998

[25] Raspor P. Faces of foods on the world of food systems. Acta Aliment. 2006;35(3):247–9.

[26] da Cunha DT Stedefeldt E Luning PA Prates CB Zanin LM. Food safety culture as a behavioural phenomenon shaping food safety. Curr Opin Food Sci. 2025;63:101305. 10.1016/j.cofs.2025.101305

[27] Marsden T Banks J Bristow G. Food supply chain approaches: Exploring their role in rural development. Sociol Ruralis. 2000;40(4):424–38. 10.1111/1467-9523.00158

[28] Yiannas F. Food safety = behavior. 30 proven techniques to enhance employee compliance. New York, NY, USA: Springer; 2015. 10.1007/978-1-4939-2489-9

[29] Griffith CJ. Food safety: Where from and where to? Br Food J. 2006;108(1):6–15. 10.1108/00070700610637599

[30] Jevšnik M Kirbiš A Vadnjal S Jamnikar-Ciglenečki U Ovca A Kavčič M. Food safety knowledge among professional food handlers in Slovenia: The results of a nation-wide survey. Foods. 2023;12(14):2735. 10.3390/foods12142735 37509827 PMC10379724

[31] Jevšnik M Raspor P. The human factor as a trigger of food safety culture within food networks: The review. Acta Microbiol Bulg. 2020;36(4):115–31.

[32] Redmond EC Griffith CJ Slader J Humphrey TJ. Microbiological and observational analysis of cross contamination risks during domestic food preparation. Br Food J. 2004;106(8):581–97. 10.1108/00070700410553585

[33] Clayton DA Griffith CJ Price P Peters AC. Food handlers’ beliefs and self-reported practices. Int J Environ Health Res. 2002;12(1):25–39. 10.1080/09603120120110031 11970813

[34] Jevšnik M Hlebec V Raspor P. Food safety knowledge and practices among food handlers in Slovenia. Food Control. 2008;19(12):1107–18. 10.1016/j.foodcont.2007.11.010

[35] Ko WH. The relationships among professional competence, job satisfaction and career development confidence for chefs in Taiwan. Int J Hospit Manag. 2012;31(3):1004–11. 10.1016/j.ijhm.2011.12.004

[36] Raspor P Jevšnik Podlesnik M Kirbiš A Raspor Lainšček P Ambrožič M. Food safety between Scylla and Charybdis - Between the European Food Safety Authority and the consumer. Med Razgl. 2012;51 Suppl 6:25–33.

[37] Jevšnik M Hlebec V Raspor P. Meta-analysis as a tool for barriers identification during HACCP implementation to improve food safety. Acta Aliment. 2006;35(3):319–53. 10.1556/AAlim.35.2006.3.9

[38] Henson S, Humphrey J. The impacts of private food safety standards on the food chain and on public standard-setting processes. Geneva, Switzerland: Food and Agriculture Organization of the United Nations and World Health Organization (FAO/WHO); 2009. Available from: https://www.fao.org/4/i1132e/i1132e00.pdf.

[39] Henson S Reardon T. Private agri-food standards: Implications for food policy and the agri-food system. Food Policy. 2005;30(3):241–53. 10.1016/j.foodpol.2005.05.002

[40] Clayton DA Griffith CJ. Observation of food safety practices in catering using notational analysis. Br Food J. 2004;106(3):211–27. 10.1108/00070700410528790

[41] Manning L. Food safety and brand equity. Br Food J. 2007;109(7):496–510. 10.1108/00070700710761491

[42] Raspor P, Jevšnik M. Food supply chains *vs* food supply nets. In: Nedović V, Raspor P, Lević J, Tumbas Šaponjac V, Barbosa-Cánovas G, editors. Emerging and traditional technologies for safe, healthy and quality food. Food Engineering Series. Cham, Switzerland: Springer; 2016. pp. 9-32. 10.1007/978-3-319-24040-4_2

[43] Safeguarding our agrifood systems: A One Health approach to food safety. Geneva, Switzerland: Food and Agriculture Organization of the United Nations (FAO); 2020. Available from: https://www.fao.org/one-health/areas-of-work/food-safety/en.

[44] A culture of food safety: A position paper from the Global Food Safety Initiative (GFSI). The Consumer Goods Forum; 2018. Available from: https://www.mygfsi.com/wp-content/uploads/2019/09/GFSI-Food-Safety-Culture-Full.pdf.

[45] Raspor P. Expert knowledge elicitation: Training programme/course on evidence collection, management and integration. Rome, Italy: BTSF Academy; 2025. Available from: https://better-training-for-safer-food.ec.europa.eu/training/course/info.php?id=331&lang=en.

[46] Wilson AM Meyer SB Webb T Henderson J Coveney J McCullum D How food regulators communicate with consumers about food safety. Br Food J. 2015;117(8):2129–42. 10.1108/BFJ-12-2014-0419

[47] Antle JM. Benefits and costs of food safety regulation. Food Policy. 1999;24:605–23. 10.1016/S0306-9192(99)00068-8

[48] Chapman B Eversley T Fillion K MacLaurin T Powell D. Assessment of food safety practices of food service food handlers (risk assessment data): Testing a communication intervention (evaluation of tools). J Food Prot. 2010;73(6):1101–7. 10.4315/0362-028X-73.6.1101 20537267

[49] Nordhagen S Lee J Onuigbo-Chatta N Okoruwa A Monterrosa E Lambertini E What is safe and how much does it matter? Food vendors’ and consumers’ views on food safety in urban Nigeria. Foods. 2022;11(2):225. 10.3390/foods11020225 35053957 PMC8774326

[50] Bozkurt H Phan-Thien KM van Ogtrop F Bell T McConchie R. Outbreaks, occurrence, and control of norovirus and hepatitis a virus contamination in berries: A review. Crit Rev Food Sci Nutr. 2021;61(1):116–38. 10.1080/10408398.2020.1719383 32008374

[51] Olaimat AN Taybeh AO Al-Nabulsi A Al-Holy M Hatmal MM Alzyoud J Common and potential emerging foodborne viruses: A comprehensive review. Life. 2024;14(2):190. 10.3390/life14020190 38398699 PMC10890126

[52] Ropkins K Beck AJ. Evaluation of worldwide approaches to the use of HACCP to control food safety. Trends Food Sci Technol. 2000;11(1):10–21. 10.1016/S0924-2244(00)00036-4

[53] Redmond EC Griffith CJ. Consumer perceptions of food safety risk, control and responsibility. Appetite. 2004;43(3):309–13. 10.1016/j.appet.2004.05.003 15527934

[54] De Boeck E Jacxsens L Bollaerts M Uyttendaele M Vlerick P. Interplay between food safety climate, food safety management system and microbiological hygiene in farm butcheries and affiliated butcher shops. Food Control. 2016;65:78–91. 10.1016/j.foodcont.2016.01.014 26630748

[55] Yeargin TA Gibson KE Fraser AM. New approach to food safety training: A review of a six-step knowledge-sharing model. J Food Prot. 2021;84(11):1852–62. 10.4315/JFP-21-146 34129677

[56] Jevšnik M Pirc L Ovca A Šantić M Raspor P Godič Torkar K. A multimethod study on kitchen hygiene, consumer knowledge and food handling practices at home. Processes. 2022;10(10):2104. https://www.mdpi.com/2227-9717/10/10/2104 10.3390/pr10102104

[57] Fatimah UZAU Strohbehn CH Arendt SW. An empirical investigation of food safety culture in onsite foodservice operations. Food Control. 2014;46:255–63. 10.1016/j.foodcont.2014.05.029

[58] Sneed J Strohbehn CH. Trends impacting food safety in retail foodservice: Implications for dietetics practice. J Am Diet Assoc. 2008;108(7):1170–7. 10.1016/j.jada.2008.04.009 18589025

[59] Howells AD Roberts KR Shanklin CW Pilling VK Brannon LA Barrett EB. Restaurant employees’ perceptions of barriers to three food safety practices. J Am Diet Assoc. 2007;107(8):A73. 10.1016/j.jada.2007.05.181 18656574

[60] Yeargin TA Gibson KE Fraser AM. New approach to food safety training: A review of a six-step knowledge-sharing model. J Food Prot. 2021;84(11):1852–62. 10.4315/JFP-21-146 34129677

[61] Strohbehn C Sneed J Paez P Meyer J. Hand washing frequencies and procedures used in retail food services. J Food Prot. 2008;71(8):1641–50. 10.4315/0362-028X-71.8.1641 18724759

[62] Webb M Morancie A. Food safety knowledge of foodservice workers at a university campus by education level, experience, and food safety training. Food Control. 2015;50:259–64. 10.1016/j.foodcont.2014.09.002

[63] Strohbehn C Shelley M Arendt S Correia AP Meyer J Ungku Fatimah UZA Retail foodservice employees’ perceptions of barriers and motivational factors that influence performance of safe food behaviors. Food Prot Trends. 2014;34(3):139–50.

[64] De Boeck E Jacxsens L Goubert H Uyttendaele M. Ensuring food safety in food donations: Case study of the Belgian donation/acceptation chain. Food Res Int. 2017;100(Part 2):137–49. 10.1016/j.foodres.2017.08.046 28888434

[65] da Cunha DT Prates CB Canuto IG Stedefeldt E Luning PA Zanin LM. The relationship of food safety culture elements: A serial mediation model. Food Control. 2025;169:111022. 10.1016/j.foodcont.2024.111022

[66] Smigic N Djekic I Martins ML Rocha A Sidiropoulou N Kalogianni EP. The level of food safety culture in food establishments in three European countries. Food Control. 2016;63:187–94. 10.1016/j.foodcont.2015.11.017

[67] Ncube F Kanda A Chijokwe M Mabaya G Nyamugure T. Food safety knowledge, attitude and practices of restaurant food handlers in a lower-middle-income country. Food Sci Nutr. 2020;8(3):1677–87. 10.1002/fsn3.1454 32180975 PMC7063367

[68] Clayton DA Griffith CJ. Efficacy of an extended theory of planned behaviour model for predicting caterers’ hand hygiene practices. Int J Environ Health Res. 2008;18(2):83–98. 10.1080/09603120701358424 18365799

[69] Raspor P. The European declaration on food, technology and nutrition. Acta Aliment. 2009;38(1):3–7. 10.1556/AAlim.38.2009.1.2

[70] Canuto IG Montezano de Carvalho IM Buarque PR Soon-Sinclair JM Stedefeldt E Zanin LM Does physical structure drive food safety behaviors of food handlers? A broken window theory perspective. Food Control. 2025;178:111464. 10.1016/j.foodcont.2025.111464

[71] Raspor P. Safety, food security and health. In: Raspor P, editor. World Food Safety Day 2020: 2nd Conference on World Food Safety Day 2020, Ljubljana, 8 June 2020. Ljubljana, Slovenia: National Council of the Republic of Slovenia; Administration for Food Safety, Veterinary and Plant Protection; 2020. pp. 13-4. Available from: https://peter-raspor.eu/wp-content/uploads/2023/05/00-koncno-WFSD-2020s2.pdf.

[72] Alkhaldi S Hod R Md Isa Z Idris IB Karim N. The impact of food safety training programs on knowledge, attitude, and practice on food safety among migrant workers – A review. Curr Res Nutr Food Sci. 2025;13(2):541–58. 10.12944/CRNFSJ.13.2.1

[73] Lee HK Abdul Halim H Thong KL Chai LC. Assessment of food safety knowledge, attitude, self-reported practices, and microbiological hand hygiene of food handlers. Int J Environ Res Public Health. 2017;14(1):55. 10.3390/ijerph1401005528098788

[74] Zheng M Bai L Liu C Gong S. Improving household food safety through combining nudge strategies with knowledge-based intervention: The effectiveness and mechanism. Food Control. 2025;168:110923. 10.1016/j.foodcont.2024.110923

[75] Sartoni M Semercioz Oduncuoglu AS Guidi A Annosi MC Luning PA. Towards digitalisation of food safety management systems - enablers and constraints. Food Control. 2025;168:110952. 10.1016/j.foodcont.2024.110952

[76] Ovca A Jevšnik M Raspor P. Curriculum analysis of food safety competences at elementary and upper-secondary level of formal education inside food-related programmes in Slovenia. J Food Sci Educ. 2018;17(2):42–51. 10.1111/1541-4329.12136

[77] Štefančič V Jevšnik M. Nudge tools for improving hygiene behavior among food handlers: Case study. J Food Saf. 2020;40(5):e12836. 10.1111/jfs.12836

[78] Jevšnik M. Nudge tools: Are they effective to improve hygiene behavior of food handlers? J Food Technol Pres. 2021;5(3):1–4.

[79] Reisch LA. Shaping healthy and sustainable food systems with behavioural food policy. Eur Rev Agric Econ. 2021;48(4):665–93. 10.1093/erae/jbab024

[80] Lindstrom KN Tucker JA McVay M. Nudges and choice architecture to promote healthy food purchases in adults: A systematized review. Psychol Addict Behav. 2023;37(1):87–103. 10.1037/adb0000892 36395010

[81] Iranmanesh M Zailani S Hyun SS Ali MH Kim K. Impact of lean manufacturing practices on firms’ sustainable performance: Lean culture as a moderator. Sustainability. 2019;11(4):1112. 10.3390/su11041112

[82] Tortorella GL Powell D Hines P Cawley Vergara AM Tlapa-Mendoza D Vassolo R. How does artificial intelligence impact employees’ engagement in lean organisations? Int J Prod Res. 2025;63(3):1011–27. 10.1080/00207543.2024.2368698

[83] Verma S Singh V. Impact of artificial intelligence-enabled job characteristics and perceived substitution crisis on innovative work behavior of employees from high-tech firms. Comput Human Behav. 2022;131:107215. 10.1016/j.chb.2022.107215

[84] Bánáti D, Jevšnik M, Nyambayo I, Bogueva D, Stanley NL. Consumer perception of food safety in Europe. In: Bogueva D, editor. Consumer perceptions and food. Singapore: Springer; 2024. pp. 415-55. 10.1007/978-981-97-7870-6_21

[85] Pai AS Jaiswal S Jaiswal AK. A comprehensive review of food safety culture in the food industry: Leadership, organizational commitment, and multicultural dynamics. Foods. 2024;13(24):4078. 10.3390/foods13244078 39767017 PMC11675119

[86] Nickell GS Hinsz VB. Applying the theory of planned behavior to understand workers’ production of safe food. Eur J Work Organ Psychol. 2023;39(2):89–100. 10.5093/jwop2023a10

[87] Manning L. The influence of organizational subcultures on food safety management. J Mark Channels. 2017;24(3-4):180–9. 10.1080/1046669X.2017.1393235

[88] Trienekens J Zuurbier P. Quality and safety standards in the food industry, developments and challenges. Int J Prod Econ. 2008;113(1):107–22. 10.1016/j.ijpe.2007.02.050

[89] Havinga T. Private regulation of food safety by supermarkets. Law Policy. 2006;28(4):515–33. 10.1111/j.1467-9930.2006.00237.x

[90] Mlakar T Mihelič Zajec A Jevšnik M. Use of a nudge tool for improving hand hygiene in a nursing team in home for elderly people: Case study. Int J Sanitary Eng Res. 2017;11(1):33–46.

[91] El Majjodi A Starke AD Trattner C. Integrating digital food nudges and recommender systems: Current status and future directions. IEEE Access. 2025;13:123002–17. 10.1109/ACCESS.2025.3588663

[92] Rogerson M Parry GC. Blockchain: Case studies in food supply chain visibility. Supply Chain Manag. 2020;25(5):601–14. 10.1108/SCM-08-2019-0300

[93] Vasileiou M Kyrgiakos LS Kleisiari C Lappas PZ Tsinopoulos C Kleftodimos G Digital transformation of food supply chain management using blockchain: A systematic literature review towards food safety and traceability. Bus Inf Syst Eng. 2025. 10.1007/s12599-025-00948-0

[94] Manning L Soon JM. Building strategic resilience in the food supply chain. Br Food J. 2016;118(6):1477–93. 10.1108/BFJ-10-2015-0350

[95] Henson S Humphrey J. Understanding the complexities of private standards in global agri-food chains as they impact developing countries. J Dev Stud. 2010;46(9):1628–46. 10.1080/00220381003706494 21328807

[96] Trienekens JH Wognum PM Beulens AJM van der Vorst JGAJ. Transparency in complex dynamic food supply chains. Adv Eng Inform. 2012;26(1):55–65. 10.1016/j.aei.2011.07.007

[97] Garcia Martinez M Fearne A Caswell JA Henson S. Co-regulation as a possible model for food safety governance: Opportunities for public-private partnerships. Food Policy. 2007;32(3):299–314. 10.1016/j.foodpol.2006.07.005

[98] Jevšnik M Raspor P. Food safety knowledge and behaviour among food handlers in catering establishments: A case study. Br Food J. 2022;124(10):3293–307. 10.1108/BFJ-09-2020-0795

[99] Raspor P Jevšnik M. Good nutritional practice from producer to consumer. Crit Rev Food Sci Nutr. 2008;48(3):276–92. 10.1080/10408390701326219 18274976

[100] Jespersen L Griffiths M Maclaurin T Chapman B Wallace CA. Measurement of food safety culture using survey and maturity profiling tools. Food Control. 2016;66:174–82. 10.1016/j.foodcont.2016.01.030

[101] Nyarugwe SP Linnemann A Hofstede GJ Fogliano V Luning PA. Determinants for conducting food safety culture research. Trends Food Sci Technol. 2016;56:77–87. 10.1016/j.tifs.2016.07.015

[102] Spagnoli P Defalchidu L Vlerick P Jacxsens L. The relationship between food safety culture maturity and cost of quality: An empirical pilot study in the food industry. Foods. 2024;13(4):571. 10.3390/foods13040571 38397548 PMC10887550

